# Towards early diagnosis of Alzheimer’s disease: advances in immune-related blood biomarkers and computational approaches

**DOI:** 10.3389/fimmu.2024.1343900

**Published:** 2024-04-23

**Authors:** Sophia Krix, Ella Wilczynski, Neus Falgàs, Raquel Sánchez-Valle, Eti Yoles, Uri Nevo, Kuti Baruch, Holger Fröhlich

**Affiliations:** ^1^Department of Bioinformatics, Fraunhofer Institute for Algorithms and Scientific Computing (SCAI), Sankt Augustin, Germany; ^2^Bonn-Aachen International Center for Information Technology (b-it), University of Bonn, Bonn, Germany; ^3^Department of Biomedical Engineering, The Iby and Aladar Fleischman Faculty of Engineering, Tel Aviv University, Tel Aviv, Israel; ^4^Alzheimer’s Disease and Other Cognitive Disorders Unit, Neurology Service, Hospital Clínic de Barcelona, Fundació de Recerca Clínic Barcelona-Institut d'Investigacions Biomèdiques August Pi i Sunyer (FCRB-IDIBAPS), University of Barcelona, Barcelona, Spain; ^5^ImmunoBrain Checkpoint Ltd., Rechovot, Israel; ^6^Sagol School of Neuroscience, Tel Aviv University, Tel Aviv, Israel

**Keywords:** Alzheimer’s disease, biomarkers, early diagnosis, blood-based biomarker, immune system, modeling, agent-based modeling, machine learning

## Abstract

Alzheimer’s disease has an increasing prevalence in the population world-wide, yet current diagnostic methods based on recommended biomarkers are only available in specialized clinics. Due to these circumstances, Alzheimer’s disease is usually diagnosed late, which contrasts with the currently available treatment options that are only effective for patients at an early stage. Blood-based biomarkers could fill in the gap of easily accessible and low-cost methods for early diagnosis of the disease. In particular, immune-based blood-biomarkers might be a promising option, given the recently discovered cross-talk of immune cells of the central nervous system with those in the peripheral immune system. Here, we give a background on recent advances in research on brain-immune system cross-talk in Alzheimer’s disease and review machine learning approaches, which can combine multiple biomarkers with further information (e.g. age, sex, APOE genotype) into predictive models supporting an earlier diagnosis. In addition, mechanistic modeling approaches, such as agent-based modeling open the possibility to model and analyze cell dynamics over time. This review aims to provide an overview of the current state of immune-system related blood-based biomarkers and their potential for the early diagnosis of Alzheimer’s disease.

## 1 Introduction

Alzheimer’s disease (AD) is prone to have a strongly increasing prevalence worldwide due to the aging society. The number of individuals living with AD is estimated to triple from 2019 to 2050, rising from 57.4 million to 152.8 million (1). Deaths due to AD have increased in recent years, making AD one of the top leading causes of death in many countries around the globe (2–4). The onset of the disease likely starts decades before the first symptoms of cognitive decline appear, when changes in amyloid-beta (Aβ) or tau in the cerebrospinal fluid (CSF) as well as in positron emission tomography (PET) measurements become detectable (5). According to the Amyloid/Tau/Neurodegeneration (ATN) framework (6), levels of Aβ_42_/Aβ_40_ ratio, phospho-tau (pTau) and neurofilament light (Nfl) in the CSF determine the diagnosis of AD biologically. PET neuroimaging and/or CSF-based measurements are both costly or invasive procedures that are only performed at specialized centers with long waiting lists. On the other hand, screening cognitive tests available at primary care are frequently not sensitive enough to identify the disease in the stage of mild cognitive impairment (MCI). In conclusion, there are currently limited resources for an early diagnosis of AD.

This situation contrasts with the requirements of recently available treatment options, in particular aducanumab and lecanumab (7, 8), which received regulatory approvals by the US Food and Drugs Administration (FDA) in 2021 and 2023, respectively. Their effectiveness has only been demonstrated in individuals with early AD, diagnosed with mild MCI or mild dementia due to AD. Given that these treatments are only beneficial in patients in an early stage of AD, it is unclear how a larger fraction of patients could benefit from the novel therapeutic options within the current healthcare setting. It is thus essential to come up with easy to use and reliable diagnostic procedures that could help to identify subjects at early clinical stages.

One option in this regard are blood-based surrogates of Aβ and pTau which have been developed in recent years. Plasma levels of Aβ_42_/Aβ_40_ ratio, P-tpTau181 and neurofilament light in the blood were found to reliably predict the risk for developing dementia due to AD in cognitively unimpaired populations, which would be beneficial for designing cost-effective clinical trials (9). Between 2020 and 2023, several blood tests have b measuring Aβ_42/40_ levels, pTau 181 and apolipoprotein (APOE) E4 (Elecsys^®^ Amyloid Plasma Panel by Roche, PrecivityAD^®^ by C2N Diagnostics, HISCL β-Amyloid 1-42/1-40 Assay Kit by Sysmex Corporation^®^, Simoa by Quanterix^®^). First validation of the usefulness of such blood tests for patient selection were carried out in prospective studies (10–14). Efforts have been made to develop an assay to separate brain-derived tau from total tau, which showed a strong correlation of brain-derived tau levels in serum with CSF samples and achieved equivalent diagnostic performance (15). As one of the most recent developments, a test measuring plasma pTau217 showed superior or equivalent performance to traditional CSF tests in detecting tau-PET and Aβ-PET status, which was evaluated in a Swedish and a US cohort (16), and similar results were seen in another study using a commercial pTau217 assay (17).

In response to recent advancements in plasma biomarkers, the US Alzheimer’s Association has revised the criteria for diagnosing and staging Alzheimer’s disease. The updated guidelines include plasma biomarkers of Aβ and pTau as surrogates for CSF/PET measurements, as outlined in their proposal (18). Additionally, the proposed framework introduces a new category for biomarkers associated with inflammatory and immune processes, reflecting the reactivity of astrocytes and microglia. This adjustment addresses developments in recent years, highlighting the increasingly apparent cross-talk between the immune cells of the central nervous system and those in the periphery (19–24). Moreover, there has been a notable increase in research findings related to AD and immune system-related genes over the last decades, with a significant portion falling into the biomarkers category (see **Supplementary Figure 1**). Recognizing emergent plasma biomarkers as valuable extensions of the pre-existing ATN framework represents a crucial step towards a more accessible and earlier diagnosis of AD. This inclusive approach integrates evolving insights into immune system involvement with the emerging significance of plasma biomarkers, contributing to a more comprehensive diagnostic framework.

The purpose of this article is to a) shed light on the recent findings on the role of the immune system in Alzheimer’s disease, and b) to point out the connection with computational approaches, including machine learning and prospectively also mechanistic modeling, for the successful identification of immune based blood biomarkers and simulation of cell dynamics.

## 2 Brain-immune system crosstalk in Alzheimer’s disease

Recent anatomical and functional discoveries of ways by which the immune system interacts with the brain have shaped the long-held dogma of the brain as an immune privileged organ. Instead of being considered as solely secluded behind the blood-brain-barrier from having any interaction with the peripheral immune system, it is becoming increasingly clear that the central nervous system (CNS) is in constant dialogue with immune cells, and that these interactions are taking place at unique anatomical barriers, such as the choroid plexus (22, 25) and the meningeal spaces (26, 27).

This life-long crosstalk shapes brain function in health and disease and was repeatedly implicated in AD development and progression (28). For example, various studies in transgenic mouse models of AD have shown that increasing recruitment of myeloid cells to the brain is associated with reduced amyloid pathology and improved cognitive performance (29–34). While the mechanism of action is not fully understood, it seems that bone-marrow derived myeloid cells that enter the brain perform distinct roles, compared to the local microglia, in reducing neuroinflammation and toxic protein pathology.

In the event of CNS injury, the neuroinflammation signal cascade is triggered, with the aim of protecting neuronal structure, preserving neuronal function, and repairing damage in affected tissues. The activation of glial cells marks the starting point for the neuroinflammatory process through the release of cytokines and growth factors. Glial cells are abundant in the brain, and exhibit diverse characteristics, functions, and phylogenetic origins. Some, such as astrocytes (of neural origin) and microglia (differentiated blood monocytes), play prominent roles in maintaining homeostasis and participating in neuroinflammatory processes (35).

Nevertheless, despite the initial positive effect of neuroinflammation on the post-injured tissue, this mechanism can eventually become detrimental to neuronal homeostasis and associated processes (36). Chronic or imbalanced inflammatory responses, driven by aging, may contribute to and perpetuate the physiopathology of neurodegenerative diseases, including AD. Although the specific immune cross-talk between microglia, astrocytes, and neurons is still a matter of discussion, many studies suggested that the neuroprotective mechanisms of glia/astrocytes turn neurotoxic by interacting with Aβ promoting senile plaques and tau accumulation through a cascade of pro-inflammatory mediators. These include the release of nitric oxide and cytokines, which eventually contribute to neuronal death. Due to this cascade of astrocytic and microglial activation, immune-derived molecules are released, allowing for their measurement as specific soluble markers for astrocytic and microglial activity.

### 2.1 Immune cellular biomarkers

#### 2.1.1 Microglia

Microglia, the resident immune cells of the brain, are derived from monocyte precursor cells and play a central role in immune defense and maintenance of brain homeostasis. They are the first line of defense against invading pathogens and respond to injury or disease states by becoming activated and performing tasks such as clearance of cellular debris and dead cells.

Microglia have been shown to play a dual role in AD. In the early stages, microglia can limit Aβ accumulation by phagocytosing these peptides and promoting their clearance. This neuroprotective role of microglia is substantiated by numerous studies demonstrating that stimulation of microglial activity can result in reduced Aβ burden and improved cognitive function (37). However, prolonged activation of microglia can lead to a chronic inflammatory state, causing neurotoxicity and contributing to neuronal death (38). Changes in expression profiles and morphology of microglia have been observed in presence of Aβ aggregates (39–43). Indeed, microglial activation is a common feature in AD brains, and is often associated with increased levels of pro-inflammatory cytokines (44). Measuring microglial activity using soluble markers has gained interest in the field of AD biomarker identification, where Triggering receptor expressed on myeloid cells 2 (TREM2) and Galectin-3 (Gal-3) are the most studied soluble markers.

##### 2.1.1.1 Triggering receptor expressed on myeloid cells 2 (TREM2)

The receptor TREM2 is expressed on the surface of microglia and mediates interactions with Aβ. TREM2 regulates Aβ degradation and clearance by binding to Aβ and bringing it to the microglia’s lysosome (45). A loss of function of TREM2 in microglia leads to a decreased microglial clustering and increased Aβ seeding, indicating a major role in development of Aβ pathology. The expression of TREM2 in AD could change the response of microglia to Aβ. Recent evidence in individuals with and without AD pathology showed that increased levels of CSF TREM2 were associated with the slower amyloid accumulation, lower levels of tau (measured by PET scans) and cognitive decline, highlighting the protective functions of microglial in AD (46, 47). Soluble TREM2 (sTREM2) levels in the CSF are highly correlated with plasma sTREM2 (48). Peripheral (plasma) sTREM2 is increasingly altered in progressive stages of AD (48, 49). Moreover, lower TREM2 levels are associated with changes in peripheral immune response in AD, including other inflammatory factors such as fibroblast growth factor-2, GM-CSF, or IL-1β (49).

##### 2.1.1.2 Galectin-3 (Gal-3)

Galectin-3 (Gal-3) is a beta-galactosidase binding protein involved in microglial activation. In contrast to TREM2, Gal-3 seems to have a deleterious role in AD. It is primarily expressed around Aβ plaques in both human and mouse brains and knocking out Gal-3 reduces AD pathology in AD-model mice (50). Compared to controls, CSF Gal-3 levels are elevated in AD patients and correlate with tau and synaptic markers (GAP-43 and neurogranin) instead of Aβ. In addition, it is associated with other CSF neuroinflammatory markers, including sTREM-2, GFAP, and YKL-40 (51). Studies including CSF and serum measurements of Gal-3 in AD or other neurodegenerative diseases showed similar results (52). Moreover, Gal-3 levels are progressively increased across the AD stage and are associated with reduced global cognitive outcomes (MMSE) (53). Plasma levels of Gal-3 have been reported to be increased in individuals with AD and might serve as a future plasma biomarker in the early stages of the disease (54).

#### 2.1.2 Astrocytes

Astrocytes, the most abundant glial cells in the central nervous system, derive from neural stem cells and play a crucial role in maintaining homeostasis. They provide metabolites and growth factors to neurons, are pivotal in synapse formation and plasticity, and regulate the extracellular balance of ions while removing free radicals. However, under pathological conditions, astrocytes undergo morphological and functional changes, leading to cell hypertrophy (reactive astrocytes) and an increased release of neurotoxic factors.

In AD, reactive astrocytes aggregate in the vicinity of Aβ plaques, as post-mortem and rodent studies showed, indicating a direct interaction between Aβ and astrocytes (55). The activation of astrocytes also modifies protein expression, including the glial fibrillary acidic protein (GFAP) and chitinase-3-like protein 1 (YKL-40), both soluble astrocytic markers typically measured in patients with AD.

##### 2.1.2.1 Glial fibrillary acidic protein (GFAP)

A significant increase in GFAP levels measured by CSF and plasma has been reported in AD compared to control individuals even in preclinical stages of the disease (56, 57). Importantly, clinical studies support that plasma measures of GFAP correlate with its CSF levels, suggesting that plasma measures are a robust proxy of astrocyte reactivity in the brains of living individuals (58). In addition, it has been shown that GFAP levels in both CSF and plasma correlate with cognitive and biological measures of AD progression. For instance, elevated plasma and GFAP levels are associated with lower cognitive performance and steeper cognitive decline (59, 60). Moreover, recent studies have shown that higher GFAP plasma levels correlate particularly with amyloid burden measured both by CSF and amyloid-PET quantification in opposition to Tau burden (46, 61, 62). Furthermore, Aβ pathology has been associated with increased plasma pTau levels only in individuals positive for astrocyte reactivity (i.e., elevated GFAP), suggesting a modulating role of astrocytic activity between Aβ and tau pathology in AD (63).

##### 2.1.2.2 Chitinase-3-like protein 1 (CHI3L1/YKL-40)

YKL-40 is a glycoprotein that is secreted by astrocytes in neuroinflammatory condition (64). A meta-analysis including 14 cohorts demonstrated a significant increase in YKL-40 CSF levels in AD compared to cognitively unimpaired controls (65). It has also been reported to be indicative of individuals with MCI and prodromal AD (66, 67). A recent study showed that YKL-40 was positively associated with memory performance and negatively associated with brain Aβ deposition, suggesting a potentially protective effect of glia on incipient brain Aβ accumulation and neuronal homeostasis (68). Plasma levels of YKL-40 were shown to correlate with CSF levels and were elevated in AD individuals (69, 70).

#### 2.1.3 Monocytes

The role of infiltrating monocytes, another key component of the myeloid cell lineage, has also been explored in the context of AD. Recent evidence suggests that these cells can be recruited to the brain in response to Aβ deposition, where they differentiate into macrophages and contribute to Aβ clearance (71). Interestingly, monocytes appear to be more efficient at clearing Aβ than resident microglia (72), which may be due to their phagocytic capabilities. Indeed, cell surface marker analysis of myeloid cells that enter the brain, in comparison to microglia, show distinct expression of scavenger receptors, such as MSR1 (73), and ability to phagocytose Aβ plaques (74, 75) or reduce soluble Aβ oligomer pathology (76). Another intriguing aspect of myeloid cell function in AD is their potential role in tau pathology. Tau is a microtubule-associated protein that becomes hyperphosphorylated and forms neurofibrillary tangles in AD. Recent studies in tauopathy mouse models have shown that recruitment of blood monocytes to the brain is associated with a reduction in tau pathology (33, 34). However, the precise mechanism by which these cells influence tau pathology is still not well understood, and further studies are needed to elucidate their role. Particularly, it is not clear if the beneficial effect is directly mediated by the recruited myeloid cells, or an indirect effect of the reduction in neuroinflammation.

#### 2.1.4 T cells

T cells, a key component of the adaptive immune system, represent another layer of complexity in the brain-immune crosstalk in AD. The brain was thought to be devoid of T cells under non-pathological conditions, but recent studies have challenged this notion. It has been discovered that T cells are present in the meningeal spaces and choroid plexus under normal conditions and were suggested to play a role in brain function (77–79). Immunophenotyping analysis of blood samples from AD patients have shown significant reduction in T cell frequencies (80), and a recent meta-analysis of 36 studies showed that this reduction is associated with increased CD4/CD8 T cell ratio in patients with AD compared to HCs (81). Beyond these general changes in lymphocytes, consistent findings have shown changes in numbers and phenotype of various T cell subsets in AD (82).

One intriguing line of research is investigating the role of T cells in AD pathology. T cell presence was repeatedly demonstrated in post-mortem human brain tissue of persons with AD (83, 84), and in the cerebrospinal fluid (85, 86). Outside the brain, peripheral T cells in the blood show reduced frequencies in AD patients (81). In mice, brain infiltration of T cells was studied in the context of their spatial distribution in the CNS-borders, such as the meninges, and brain parenchyma, and was shown to correlate with the degree of Tau pathology and to contribute to neurotoxicity (87). However, the role of T cells in promoting or suppressing AD pathology is unclear, and most likely involves different subsets of T cells. Indeed, T cell deficiency in AD mice was shown to inhibit hippocampal neurogenesis and restrict hippocampal neuronal regeneration (88).

#### 2.1.5 Immune checkpoint PD-1/PD-L1

Of potential interest is immune checkpoint molecules as cellular biomarkers in AD. Programmed cell death protein 1 (PD-1) and its ligand PD-L1 are key immune checkpoint molecules that play a crucial role in regulating immune responses. They are typically expressed on the surface of T cells and other immune cells and serve to dampen immune responses, preventing autoimmunity and maintaining self-tolerance. Expression of PD-1 on T cells and PD-L1 on monocytes and macrophages significantly decreases in AD patients and in patients with MCI compared with age- and sex-matched healthy controls (89). In a recent study, this change in PD-1/PD-L1 expression on T cells was correlated with the different stages of AD (90). PD-1 expression was also found to increase in T cells in the CSF of AD patients (91). Beyond the potential use of PD-1/PD-L1 as cellular biomarkers in AD, this immune checkpoint pathway was also suggested as a target for therapeutic intervention. In different mouse models of AD, transient blockade of PD-1/PD-L1 resulted in reduced brain pathology and improved cognitive performance (33, 92). The mechanism of action was shown to involve homing of specialized immune cells to the brain (both myeloid cells and regulatory T cells), where these cells mitigate different pathomechanisms, ultimately leading to function improvement (34, 93).

### 2.2 Current and future potential biomarkers for MCI and AD

Since the establishment of CSF and imaging biomarkers of amyloid beta and tau in 2011 by the National Institute on Aging (NIA) and the Alzheimer’s Association (AA) (94–96), and the resulting profiling via the A/T/N framework in 2018 (6), no major changes in the official clinical criteria for diagnosis and staging of AD have been made. In 2023, the AA responded to the recent developments in plasma biomarker research and advocated the inclusion of plasma biomarkers for the diagnosis and staging of AD (18). Their current draft focuses on the inclusion of plasma surrogates for established CSF biomarkers, such as p-tau 217/np-tau 21, p-tay 205 and Nfl. Plasma p-tau 231, p-tau 181 and Aβ42/40 were not included in the current revision since these assays have not yet proven to be as accurate for diagnosis as approved CSF assays. Importantly, the AA has suggested adding a new biomarker category incorporating inflammatory and immune mechanisms (I) to the A/T/N framework. Interestingly, the original 2018 framework called for integrating biomarkers beyond the established biomarkers as a novel X category in an ATX(N) framework. They propose to include GFAP as a marker for astrocyte inflammation, measured in either CSF or plasma. This means that for the first time in clinical diagnosis, an inflammatory plasma marker could be used for staging and prognosis of AD.

**Figure 1:**
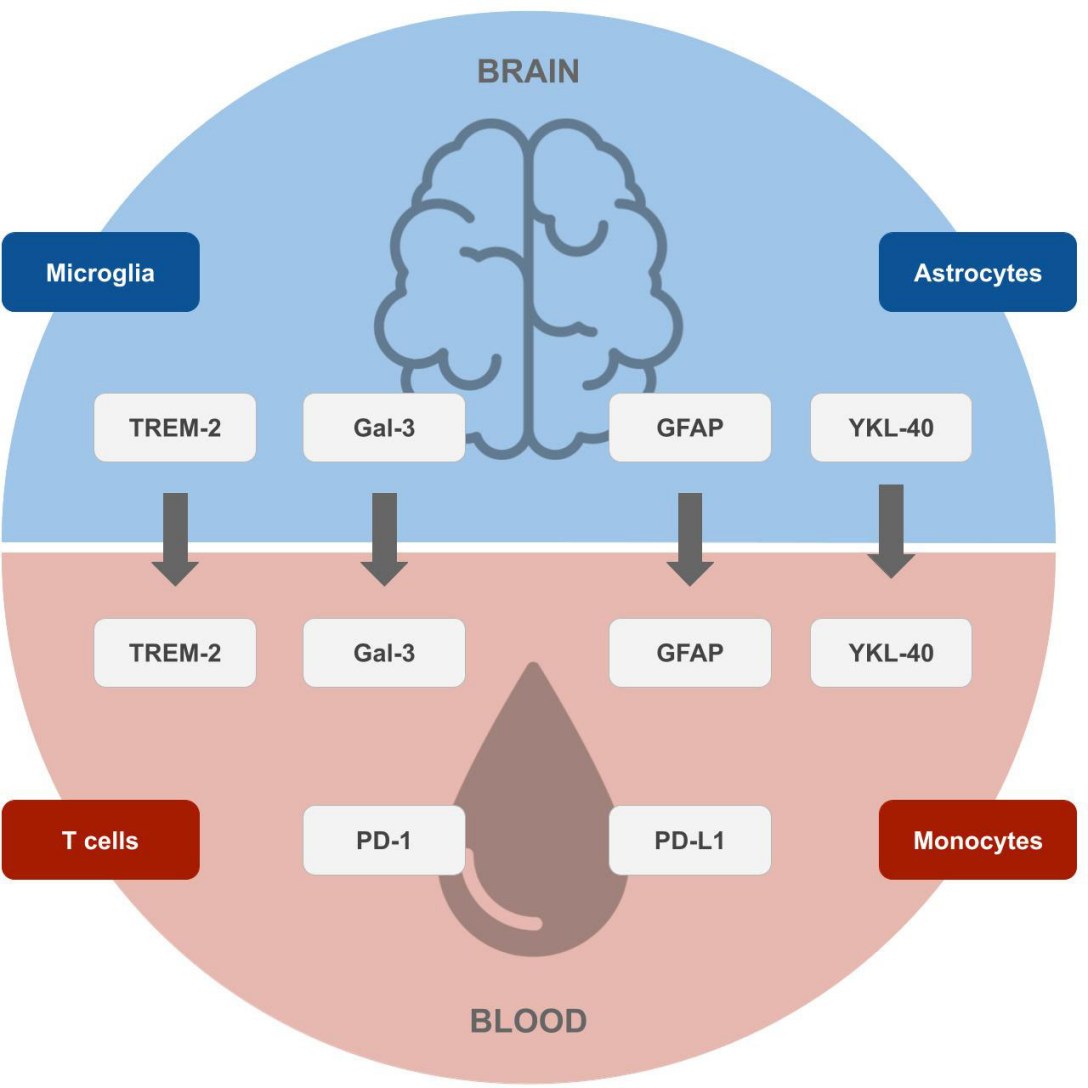
Potential future biomarkers for the early diagnosis of Alzheimer’s disease. Biomarkers of microglia and astrocytes originating in the brain (top), which correlate with surrogates in the blood, and markers of T cells and monocytes originating in the blood (bottom), which can be measured by blood tests. Designs from Freepik were used for this image (www.freepik.com).

Yet not only GFAP, as an inflammatory marker of astrocytes, but also other biomarkers of immune cells of the brain (microglia and astrocytes) and blood (T cells and monocytes) show changes in AD individuals and likely play a major role in AD physiopathology and could serve as potential future biomarkers (see **Figure 1**), as discussed in the sections above.

The accurate identification of an individual with MCI is essential for the early diagnosis of AD. In the following, we outline and discuss the relevance and potential of the immune-related plasma biomarkers mentioned in the previous section.

Plasma levels of sTREM2 were not found to be significantly changed in MCI compared to controls, and significantly decreased in AD compared to MCI (49, 97). While this does not allow for a diagnosis at an MCI stage, the study by Weber et al. found that the relationship of sTREM2-related inflammatory markers is already drastically changed at early AD stages, both in groups defined by clinical symptoms (MCI) and CSF biomarker categories (A+T−N− or A+T+N−). This suggests that dysfunctional peripheral TREM2-related inflammatory activity, in particular fibroblast growth factor-2, GM-CSF, and IL-1β alterations, plays a major role in early disease progression.

While CSF levels of Gal-3 have been seen to be elevated in AD individuals (52, 53, 98), plasma levels were not significantly increased in individuals with MCI (53), apart from reports in a preliminary study, where Gal-3 serum levels were found to correlate with clinical dementia rating (CDR) stages (54). Yet, since most studies indicate non-significant changes in Gal-3 levels at an MCI stage, it remains to be investigated whether peripheral Gal-3 levels rather reflect systemic inflammation than serving as a surrogate for expression in the brain and CSF.

GFAP levels have recently been reported to be predictive of conversion of MCI to AD-dementia in several studies with steeper trends of conversion for cognitively abnormal groups (99, 100) and were able to discriminate between MCI individuals who progressed to dementia and non-progressors (101). GFAP serum levels were also found to detect AD pathology in patients with MCI (99).

Plasma YKL-40 levels have not yet been shown to discriminate MCI from CN. However, elevated levels of YKL-40 in CSF were observed in individuals with MCI, prodromal and preclinical AD in multiple studies (66, 67, 69, 102, 103), and plasma and CSF levels were found to be correlated (69). Therefore, YKL-40 has potential to be used as a plasma biomarker for detecting early stages of AD but remains to be tested.

The population of PD-1 negative T regulatory cells has previously been reported to be significantly augmented in MCI individuals (104). Recently, the first report of changes in PD-1/PD-L1 expression in earlier stages of AD was published, where PD-L1 expression was upregulated in certain subsets of T cells in individuals with mild AD (CDR stage 1; CD3+CD56+ T cells and CD4+CD25+ T cells) and moderate AD (CDR stage 2; CD4+ and CD8+ T cells) (90). Further investigation and validation of these findings is needed, but the future use of the PD-1/PD-L1 checkpoint for biomarker development is not unlikely.

The immune markers we discussed here have varying potential as biomarkers for detection of early stages of AD, and further investigation is obligatory to ascertain their correlation with established CSF biomarkers in AD. More evidence is needed to gain a consensus on a combination of plasma biomarkers that yields the most accurate diagnosis at the earliest stage, especially for an inclusion in the AD clinical research framework. A non-exhaustive overview of established and potential future biomarkers together with their potential for diagnosis and staging is given in **Supplementary Table 1**. It is noteworthy that the prediction performance of each biomarker relies heavily on the dataset used in the respective study, and that reported performance metrics differed. Therefore, the prediction performance of aforementioned biomarkers is not directly comparable, and further comprehensive validation studies are needed. Nonetheless, plasma biomarkers have the huge advantage of being easily accessible through blood testing and could have a high potential to make far-reaching changes in clinical diagnosis and improve patient care.

## 3 Omics-based biomarker signature discovery and machine learning

Omics-based approaches should allow for an unbiased, data-driven discovery of novel immune-based blood-biomarker candidates in Alzheimer’s Disease. However, classical bulk RNA sequencing is limited since mixtures of various cell types are measured. Going one step further, recent single cell sequencing techniques now allow the measurement of the expression of all genes in individual cells - including immune cells in the blood. This provides a more detailed picture of a patient’s disease state at a given point in time and may help to obtain a better understanding of disease mechanisms as well as associated biomarkers in the future. For example, Xu and Jia analyzed single cell gene expression data of 3 AD and 3 controls, and their findings suggest that the peripheral adaptive immune response, mediated by T cells, is a factor in the pathogenesis of AD (105). In a study by Xiong et al. on single-cell RNA (scRNA) sequencing of AD patients, they identified B cells as a determinant of the severity of the disease. A lower number of B cells was found in the blood of AD vs healthy controls, and in a follow-up experiment in mice they found a correlation between depletion of B cells in early-stage AD models and accelerated cognitive decline as well as increased Aβ burden (106). Similar results were gathered in a study by Song et al., where additionally an increase in proportion of neutrophils as well as gene expression levels of AD-associated pathways in neutrophils were detected via a cellular deconvolution method on RNA bulk blood sequencing data (107).

The richness in information of scRNA sequencing data does not come without challenges. High dimensionality, technical noise and batch effects impose difficulties for data processing and require the use of specific computational tools for further analysis, which have been extensively developed in recent years (108–110).

**Figure 2:**
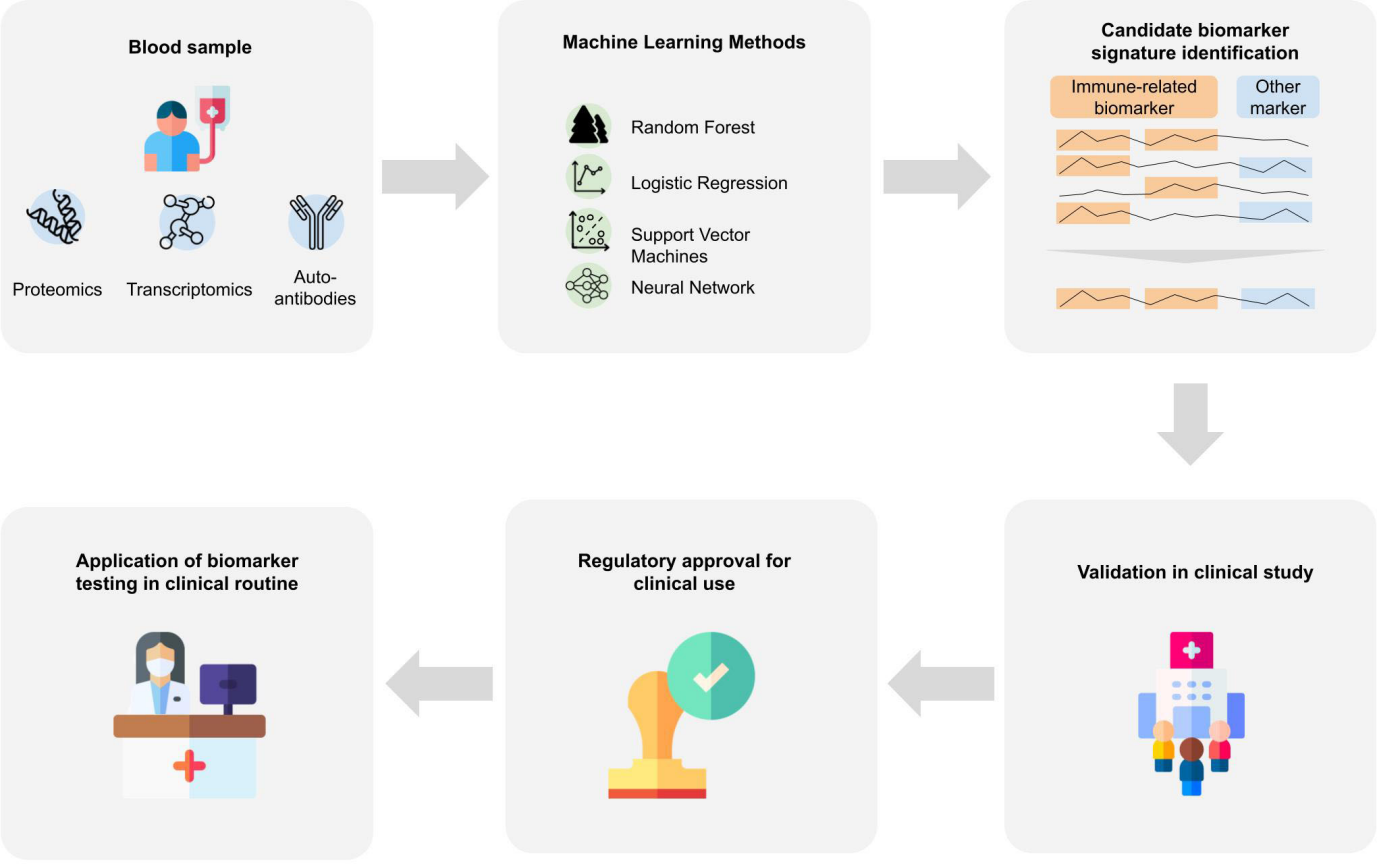
Biomarker signature identification workflows. Blood samples are taken from patients that are further analyzed to gain proteomics, transcriptomics or auto-antibody measurements. Various machine learning approaches were applied to the resulting data to derive a biomarker signature for Alzheimer’s disease. Many of the identified biomarkers which achieve good predictive performance include immune-system-related biomarkers. For an application of identified biomarkers in the clinical routine, further steps are necessary. The machine learning algorithm and the respective assays need to be validated in a prospective clinical study, and regulatory approval by national or international institutions. Designs from Freepik were used for this image (www.freepik.com).

Classically, omics data are analyzed using statistical analysis methods, which helps to provide insights into disease mechanisms and candidate biomarkers. However, statistical methods only help to understand differences between patient groups that exist on average and do not allow us to make statements about a single patient. But to support medical decisions, including early diagnosis, we need to consider and combine features of an individual patient into a diagnostic score. Since it is unlikely that a single biomarker would allow for a highly accurate identification of patients in a preclinical or prodromal stage, for this purpose machine learning (ML) plays a crucial role (111). The application of ML bears strong potential for biomarker discovery since it enables to find patterns in high dimensional data that are otherwise hard to detect. ML might help to understand the heterogeneity of the disease and aid in identifying subtype-specific biomarker signatures. In particular, precision medicine takes individual patient characteristics on a molecular level into account and can help to identify biomarker panels that allow for a precise diagnosis. In the following we review existing works focusing on the discovery of immune-based blood biomarkers signatures using machine learning. For an overview of the studies, we refer to **Supplementary Table 2**, listing approaches based on their primary data source type with information on the data set and the machine learning method. It is important to understand that bringing an ML algorithm trained on omics data into medical practice is highly complex and requires a stepwise development, validation and authorization process [(112), **Figure 2**]:

1. In addition to the original discovery dataset, external validation data should be used to better understand the generalization ability of the ML model and to detect potential biases.
2. Due to cost and efficiency reasons a customized and approved assay (e.g. by the Federal Drug and Food Administration - FDA - in the USA) for measuring biomarker candidates should be developed.
3. Further validation in a prospective clinical trial is required to demonstrate actual clinical benefit, e.g. by showing a good discrimination of MCI vs. HC and AD patients.
4. Regulatory approval as a diagnostic device and/or medical device is needed before the assay and the algorithm can be employed in medical routine.

In the following we discuss individual studies using blood-based proteomics and transcriptomics data as well as auto-antibodies in more detail.

### 3.1 Blood-based proteomics and transcriptomics approaches

In an extensive multi-center study, Morgan et al. investigated plasma biomarker candidates for the diagnosis and stratification of AD patients (113). More than 50 inflammatory proteins were measured in immunoassays, including complement components, activation products and regulators, cytokines and chemokines. A logistic regression model was trained to differentiate between the diagnosis groups enabled to identify inflammatory biomarkers that distinguished not only HC from AD, but also MCI from AD, with an AUC of 0.79 and 0.74, respectively. Plasma analytes in AD patients compared to HC were increased for C4 and eotaxin-1, and decreased for CR1, C5 and CRP. In MCI patients, increased levels of FH, C3 and MCP-1, and decreased levels of C5 and MIP-1-Beta compared to HC were found. When comparing AD to MCI, increased eotaxin-1 and MIP-1-Beta, and decreased FI, C3, CRP and MCP-1 emerged as distinguishing plasma biomarker candidates.

Prabhakar and Bhargavi investigated in a machine learning approach to identify which blood plasma proteins could be useful in the early detection of AD (114). The blood plasma protein samples were analyzed for a total of 146 protein features. The best candidate biomarker panel was identified by feature selection and subsequent combination testing with Support Vector Machines. The panel consists of the following immune-related proteins: A2M, MDC, IL-18 and CD5L. This set of candidate biomarkers achieved an accuracy ranging from 0.55 to 0.8, depending on the kernel used, for the classification of AD and HC. When applied to MCI individuals, this protein panel did not yield high accuracy results.

Karaglani et al. developed diagnostic biosignatures based on transcriptomics and proteomics from 7 public datasets for the diagnosis of AD (115). With proteomic data consisting of nearly 1000 features, they identified 7 protein candidate biomarkers using logistic regression with an AUC of 0.921, including several immune-system related proteins, such as CAMLG, IL-4, TPM1 and IL-20.

Jammeh et al. investigated the best combination of blood-based candidate biomarkers for a routine diagnosis of AD-related dementia from blood samples from the ADNI proteomic database (116). They identified a panel of candidate biomarkers, including immune-related proteins A1M, A2M, eotaxin-3, PYY, PPY and EGF via a Naïve Bayes classifier, which was able to identify AD patients with a sensitivity of 0.85 and a specificity of 0.78.

In a further study, Eke et al. tried to define an optimum panel of blood-based candidate biomarkers that can fulfill a diagnostic performance of 80% sensitivity and specificity (117). They used proteomic data from the ADNI phase 1 study. With Support Vector Machines, they could identify 5 candidate biomarkers associated with the immune response, comprising A1M, A2M, C3, and TNC, that could sufficiently distinguish AD from HC in the ADNI cohort.

Choi et al. investigated the association of protein levels of CypA, HO-1 and IRE1 in the blood with changes in gray matter volume (118). They found that in both MCI and AD individuals, blood levels of all three proteins investigated were correlated with AD signature regions of the brain. Higher CypA levels were associated with increased gray matter volume of the occipital gyrus and posterior cingulate. Gray matter volume changes at the hippocampus, uncus, lateral globus pallidus and putamen were positively associated with changes in HO-1 levels and negatively with IRE1 levels (118).

In a study by Liu et al., several subsets of immune cells were quantified in AD individuals (119). They found a significant increase in immune infiltrates in AD individuals compared to HC, such as monocytes, M0 macrophages, and dendritic cells, and a decrease in other immune cell types, such as NK cell resting, T-cell CD4 naive, T-cell CD4 memory activation, and eosinophils. They identified hub genes, which include *ABCA2*, *CREBFR*, *CD72*, *CETN2*, *KCNG1* and *NDUFA2* by applying LASSO regression and SVMs to bulk RNA gene expression profiles. A further analysis of the identified hub genes and their relation to immune factors through the TISIDB database confirmed that these genes were strongly correlated to the level of immune cell infiltration and regulators of the immune microenvironment (120).

Walker et al. developed a protein signature for dementia risk based on the dysregulation of immune and autophagy pathways in middle-aged adults (121). They analyzed 4877 plasma proteins of 10.901 individuals from the ARIC cohort in terms of their association with dementia risk up to 25 years later. They found 32 dementia-associated proteins, of which 12 were related to CSF biomarkers of AD, neurodegeneration or -inflammation. They grouped the identified plasma proteins into modules based on protein co-expression patterns and found associations of several modules with near-term or long-term dementia risk. Modules associated with long-term dementia risk were enriched for proteins involved in JAK-STAT signaling, T helper 1 and 2 cell differentiation, leukocyte activation and immune/mitogen-activated protein kinase signaling.

A study by Abdullah et al. aimed to find the transcriptomics candidate biomarkers that could most accurately classify AD patients in Malaysia (122). They used Boruta’s feature selection algorithm on a transcriptomics dataset from the TUA study, comprising 22.254 transcript genes of 92 AD patients and 92 HC. They evaluated the classification performance on several statistical and machine learning classifiers. With an elastic net logistic regression model, they achieved an accuracy of 0.82. Among the 16 potential biomarkers that they identified were ANKRD28, CCDC92, DEFA3, FBXO32, GRIA4, HDAC7, IFITM3, LY6G6D, MC1R, RPL18, SPOCD1, ST14, TOR1AIP2, TRIM16L, UBXN7, and VEGFB.

Kim and Lee proposed a pathway information-based neural network for the prediction of AD, which uses blood and brain transcriptomic signatures (123). They used pathway information from KEGG and Gene Ontology alongside gene expression data as input for their deep neural network. With the help of a backpropagation-based model interpretation method, they were able to identify essential pathways and genes in the prediction of AD. This analysis indicated an enrichment of genes involved in PI3K-Akt and MAPK signaling, two pathways which are involved in the immune response (124, 125), in association with AD.

The link between neuroinflammation and AD has gained more attention in recent years. Biological findings attribute both a detrimental as well as protective role to neuroinflammation (126). Gironi et al. proposed to use an approach that can reconstruct non-linear relationships to model the complex system of neuroinflammation. They analyzed the immunological and oxidative stress parameters in peripheral blood mononuclear of AD and MCI patients (127). They constructed a machine learning algorithm to distinguish healthy controls from AD and MCI patients and selected the most important immunological and oxidative stress parameters for the prediction. Their conclusion emphasized that the initial activation of microglia is beneficial for Aβ clearance, but the mechanism can become chronically destructive without timely control.

### 3.2 Blood-based autoantibodies

The role of autoimmunity in neurodegenerative diseases, including AD, has seen more attention recently, and offers new perspectives in terms of diagnostics and therapeutics (128–131). Autoantibodies are antibodies that react to self-antigens and are all-present in the human body. Natural autoantibodies are responsible for clearance of debris during inflammation, yet they might also amplify inflammation in systemic auto-immune and neurodegenerative diseases (132–134). Therefore, several approaches have taken up this idea and investigated blood-based autoantibodies as biomarker candidates for the diagnosis of AD.

DeMarshall et al. tried to identify biomarker candidates for patients diagnosed with MCI due to an early-stage AD pathology using autoantibodies (135). They selected 50 MCI patients and their HC from the ADNI2 study and performed protein microarrays on the serum samples to identify autoantibodies. They used a Random Forest model to identify a panel of 50 AD-associated MCI-specific biomarker candidates. They reported that their model could differentiate MCI patients from age- and gender-matched controls with a sensitivity, specificity and accuracy of 100%, and furthermore, with > 90% from mild-moderate AD. In a further study, DeMarshall et al. used this panel of autoantibody biomarker candidates on elderly hip fracture repair patients (136). With their autoantibody panel, they were able to identify the patients that were positive for CSF AD biomarkers. Recently, DeMarshall et al. proposed a multi-disease diagnostic platform based on autoantibodies to detect the presence of AD-related pathology, focusing on early stages, including the pre-symptomatic, prodromal and mild-moderate stages (137). They conclude that blood-based autoantibodies present an accurate, non-invasive, low-cost solution, especially for early diagnosis of AD in pre-symptomatic and prodromal AD stages.

## 4 Mechanistic modeling of the immune system

The immune system is dynamic and characterized by complex cell-cell interactions, which eventually manifest in the increase or decrease of certain markers over time. Therefore, purely data driven statistical and machine learning approaches, which typically rely on cross-sectional snapshot data, often lack robustness and reproducibility across studies (138). A principal alternative is thus to first come up with a detailed, quantitative understanding of fundamental disease mechanisms, from which biomarker candidates may then be derived and tested in a second step (139–141). Here, mechanistic modeling techniques could fill in a gap by simulating longitudinal data on cell-cell interactions based on parameter estimates from quantitative data. Mechanistic modeling approaches, and specifically agent-based modeling (ABM) techniques, have been developed in the past to provide a realistic simulation of mixtures of various cellular species, accurately describing cytokine concentrations, activations of cells and interactions between cellular players of the immune system over time. In the following we provide an overview about existing works focusing on the modeling of immune related mechanisms in the AD field.

### 4.1 Ordinary differential equations

Ordinary differential equations (ODEs) are widely used to mathematically describe time-dependent molecular processes in systems biology (142–150). Often experimental data obtained from cultured cells is used to define the initial conditions of such models and to infer free parameters. The ready fitted model can then be used for extrapolation of time series or for simulating counterfactual scenarios, for example the intervention by a certain drug. Learning the structure of such a mechanistic model can give biological insights and may also point towards candidate biomarkers. Recently, several mathematical models on AD progression have been published that take the role of the immune system into account, which we will elucidate in the following.

A kinetic model was used to explore the effect of microglia and astroglia on the pathogenesis of AD (151, 152). The model suggests that these immune cells promote the progression of AD via neuronal cell death. They argue that the increase in population of microglia and astroglia in AD means an increase of inflammatory cells producing toxins that eventually cause neuronal cell death. Along these lines, the aggregation of microglia in AD was proposed to be explained via a chemotaxis model (153). They modeled the interaction of several attractive and repulsive cytokines produced by microglia in order to explain under which conditions the aggregation of microglia is possible, as seen in the pathology of Alzheimer’s patients. With their model, they were able to explain the aggregation of microglia given a certain combination of chemotactic responses of microglia to IL-1beta and TNF-alpha.

Several convergent mechanisms exist in Alzheimer’s and Parkinson’s disease, including the p38 pathway activation that enhances the production of proinflammatory cytokines such as IL-1beta and TNF-alpha (154). Sasidharakurup et al. used a systems biology tool to create process diagrams of common mechanisms and converted each reaction into a mathematical equation dependent on an initial literature-derived condition. They conclude that the activation of microglia is responsible for increased levels of TNF-alpha, as observed in Alzheimer’s and Parkinson’s disease, which lead to excessive oxidative stress and result in necrosis and apoptosis (155).

Kyrtsos and Baras developed a graphical systems biology model to investigate the interaction of neuroinflammation, mitochondrial function, the ApoE genotype and A-β generation on cellular and molecular level (156). They simulated chronic low-level inflammation by increasing the level of TNF-alpha and simulated the effects on the levels of various neuroinflammatory cytokines. Interestingly, together with a triggered collapse of mitochondrial function, chronic neuroinflammation led to neuronal cell death. Their model results agree with biological findings which have shown a decreased capability of the brain to protect itself in case of chronic inflammation (157).

During the progression of AD, a strong accumulation of CD4+ T cells is seen in many patients (158). CD4+ T cells can regulate immune responses via secretion of signaling molecules, yet the set of cytokines produced by each CD4+ T cell can vary depending on the cytokines in the extracellular environment. The duration of T cell receptor engagement and co-stimulation also contributes to the differentiation of CD4+ T cells. Miskov-Zivanov et al. simulated the changes in cell fate and plasticity of CD4+ T cells with a logical circuit model (159). In their review on heterogeneity and function of CD4+ T cells, Carbo et al. gather computational approaches to model immune responses of CD4+ T cells and we refer to them for further reading (160). The relationship between antigenic stimulations of CD4+ T cells and regulatory CD4+ T cells was recently described in an ODE model (161), using their concentration and the extent of the antigenic stimulation. ODE models typically neglect spatial aspects of biophysical mechanisms. Sego et al. therefore combined non-spatial ODE modeling with spatial, cell-based modeling via a cellularization approach to create a spatiotemporal model of the immune response upon viral infection (162, 163).

Notably, most ODE approaches use initialization parameters and relative rates from the literature, hence they do not reflect individual system-level differences. These approaches can rather be seen as a generalization of immunological processes but are not suitable for patient-specific inductive reasoning.

### 4.2 Agent-based modeling and cell-cell interaction models

Cell-cell interactions are influential on organismal development and single-cell function (164). The understanding of cell-cell interactions can give insight into biological mechanisms in the development of disease. Agent-based modeling (ABM) is a powerful technique to simulate and explore phenomena that include many of active components, represented by agents. In the ABM framework the agents are operating in the system, simultaneously influencing the simulated environment and being influenced by the simulated environment. The agents can also perform actions autonomously, based on rules or state machines, with regards to their interaction with other agents and with the environment (165). These actions represent the behaviors in the real system (166–169). Modeling of a multi-scale spatiotemporal system is a complex computational challenge due to the high number of sub-processes involved, each with its own features. As the complexity of the simulated system increases in size and in the agents’ capabilities, the outcome of the simulation may reveal unpredicted emergent results that were otherwise very hard to obtain, e.g. by pure mathematical modeling (170, 171). These emergent results can be later interpreted as specific signaling pathways at the intra-cellular level thus inferring on biomarkers discovery (172). ABM requires mainly local knowledge regarding the mechanisms (rules) that govern the behavior of each type of agent and the environment, whereas global behavior emerges from the agent-agent interactions as well as the interactions with the environment.

Lately, there have been extensive research efforts to develop agent-based simulation systems. General purpose approaches include EPISIM (173, 174), SimuLife (175), CellSys (176), and several approaches have been developed to specifically model the immune system, such as the Multiscale Systems Immunology project (177), LINDSAY Composer (178), FLAME (179), Simmune (180), C-ImmSim (181) and Cell Studio (182, 183). Agent-based models tailored towards specific immune system processes also exist, such as a recently published model of adaptive immunity that describes the T cell response to various factors, including antigen-presenting dendritic cells, changes in T cell recruitment and swelling conditions of the lymph node (184).

Cell Studio (182, 183) is a unique platform for modeling complex biological systems. It provides an advanced environment specifically designed for non-coding researchers, including a visual interface, modeling of biological, biophysical, bioinformatics and chemical data, as well as parallel computing. In particular, the platform is specialized in modeling immunological response at the cellular level. Cell Studio’s main feature is to realistically model the immunological response as a multi-scale, hierarchical phenomenon that operates at molecular, cellular, tissue levels and eventually the organ level. The platform, adopting the ABM paradigm, proposes the cell as a native agent whose interactions with proteins, molecules, medium and other cells, define the main features of the immunological scenario. The choice of which interactions are necessary to describe a particular process is given to the user which can set up a unique experiment with predefined rules. To facilitate the definition process, a state-machine description of the cell is used, to control the dynamic behavior as an approximation for the intracellular processes, in line with the growing number of publicly available pathways and network databases.

Next to agent-based modeling techniques, several cell-cell communication and interaction inference approaches exist. These rely heavily on prior knowledge from pathway databases and use various methods to estimate cell-cell interactions which can be categorized into statistics-based, network-based and spatial transcriptomics-based approaches (185). Obviously, cell-cell interaction models and ABM are not necessarily separated. However, grossly speaking, cell-cell interaction models relate to networks of interactions and attempt to be very comprehensive in integrating omics data to include a multitude of possible interactions. Most ABM approaches have a similar aim, but they include additional dimensions of the interactions as not covered by most cell-cell interaction models such as space (including cellular motion, diffusion and mechanical characteristics of the medium), temporal kinetics of secretion, specific effects of different concentrations and finally the ability to include variance within same species of cells. Inclusion of this physical data comes with the price of a need to simplify the modeled interactions due to the complexity of parameterization and, later on, of computation.

Dimitrov et al. compared current cell-cell communication prediction approaches (186). Their analysis showed that immune system pathways are not equally represented in the resources used for the approaches under investigation. They found that both the interaction information resource as well as the method can considerably impact the cell-cell communication inference prediction and conclude that integration of information from additional modalities could help to refine the predictions.

Several cell-cell communication approaches have been specifically designed to integrate single-cell RNA sequencing data (164, 187), which open a new way to generate patient-specific models for cell-cell communication prediction. Since there is no ground truth for the cell-cell interaction prediction, Liu et al. suggest using spatial transcriptomics data for the evaluation of cell-cell interaction models with single-cell RNA sequencing data and provide a comparison of approaches (185).

One of the first to integrate patient-level data of Alzheimer patients into cell-cell interaction simulations was Zhao et al., using the first published single-cell RNA sequencing dataset of peripheral blood on Alzheimer’s with 3 affected individuals (105). They analyzed cell-to-cell communication networks using CellChat (188) to gain insight on signaling pathways and interactions between different cell subpopulations in Alzheimer patients as well as ovarian cancer patients (189). They found that interactions between monocytes, natural killer cells and T cells are increased in AD individuals compared to healthy controls, and that monocytes were highly influenced by HLA-related signals. They followingly designed a monocyte-based prognostic signature which was used to determine the risk of a patient and validated their risk model on ovarian cancer datasets. They successfully predicted low- and high-risk group progression and survival via Cox analysis with the risk score. Similar experiments now need to be performed on AD datasets to validate the suitability of such a monocyte-related risk model for the prediction of progression and survival of individuals affected by AD. This is one prestigious example of how cell-cell interaction models can be useful in the development of a prognostic biomarker signature based on immune signaling and cell-cell interactions.

Along these lines, simulating the cross-talk between the peripheral immune system and the brain for the condition of Alzheimer’s disease via agent-based models might open new ways to find immune-specific biomarkers that are indicative of early changes in the immune system during the onset of the disease. Monitoring changes in biomarker concentrations and expression levels during the ABM simulations might give insights into which biomarkers might be suitable to identify a patient at an early stage. Going one step further, the integration of patient-specific information as part of the initialization of the ABMs could help to shed light on the differences in biomarker levels on an individual level and thereby enable personalized predictions on the intensity and velocity of changes in biomarkers. This information could be helpful in the identification of disease mechanisms specific to the phenotype of the patient and aid in better design of patient-specific treatments.

## 5 Conclusion

Identifying patients in a preclinical or prodromal disease stage of AD is crucial for the success of currently available therapeutic options. Hence, there is an urgent need for alternative and easily accessible biomarkers which allow for an early diagnosis. Current knowledge suggests that the ATN framework for diagnosis of AD should be complemented with markers of inflammation. Considering the recently discovered cross-talk of immune cells of the central nervous system with those in the peripheral immune system, immune-based blood-biomarkers emerge as a promising option.

Data-driven biomarker discovery approaches often lack statistical robustness and reproducibility due to limited sample size and high dimensional feature space in omics data. Mechanistic modeling approaches, such as ODEs and ABMs, provide complementary methods with high statistical robustness. However, these knowledge-derived models may not fully capture the unknown aspects of the disease. Most mechanistic models have been calibrated and tested against experimental data in model systems (e.g. cell lines), but not real patient data. Recent ABMs like Cell-Studio, initialized with patient-level data such as FACS measures of cell surface markers, offer the potential for predictive results by reflecting the mixture of cell types in individual patients. In the future ABMs could incorporate single-cell RNA sequencing data to simulate gene expression dynamics. Implementing ABM platforms requires significant resources for physical and chemical parametrization, but automated literature processing using natural language processing and generative transformers may facilitate the next generation of ABM parametrization. Analyzing AB< simulations with modern data mining and machine learning techniques might uncover new perspectives for biomarker signature discovery. In particular, the consideration of a time dimension may open the possibility to identify biomarker signatures in a far more robust and statistically stable manner than currently (112).

At the same time, we would like to re-emphasize that it is unlikely that a single biomarker alone would allow for an early diagnosis with sufficiently high accuracy. Instead, the focus should be on the discovery of biomarker signatures using modern omics technology, which enables sequencing immune cells in the blood. Machine learning algorithms can then integrate signals from various genes into a single, patient-specific diagnostic score, enabling the identifying of patients at an early disease stage.

In addition to the current limitations in the capacity of the current healthcare system to thoroughly assess early stages of AD in all potential candidates within specialized memory clinics, the established diagnosis methods rely on invasive procedures for patients, such as lumbar punctures to extract CSF, or involve costly imaging procedures. These factors contribute to the challenges in providing timely and accurate diagnoses for AD patients.

Blood-based biomarker testing offers several advantages over traditional CSF-based testing. Firstly, blood tests have the potential to be more cost-effective, making them more accessible to a larger population. Additionally, they are less invasive, which reduces the discomfort and potential risks associated with invasive procedures. Moreover, blood-based biomarker testing can be performed by general practitioners, who are often the first point of contact for patients before referral to a neurologist, allowing for earlier detection and intervention.

The current diagnostic approaches for AD are predominantly confined to specialized clinics, which limits their routine use in general practice. By introducing new diagnostic procedures and providing specialized training for general practitioners and medical assistants, the use of blood-based biomarkers can be integrated into routine diagnostics. Importantly, the cost of training and adopting these new procedures is expected to be significantly lower compared to traditional diagnostic tests.

With this vision for the future, blood-based biomarkers could pave the way for a more accessible diagnostic method for AD. This, in turn, may lead to decreased healthcare costs in the long-term, more streamlined and cost-effective clinical trials, and the delivery of earlier and improved treatment options to the patients.

## Author contributions

SK: Formal analysis, Visualization, Writing – original draft, Writing – review & editing. EW: Writing – original draft, Writing – review & editing. NF: Writing – original draft, Writing – review & editing. RS-V: Funding acquisition, Writing – review & editing, Supervision. EY: Funding acquisition, Writing – review & editing, Supervision. UN: Funding acquisition, Writing – review & editing, Supervision. KB: Writing – original draft, Writing – review & editing. HF: Conceptualization, Funding acquisition, Project administration, Supervision, Writing – original draft, Writing – review & editing.
