# Supplementary material for: Towards early diagnosis of Alzheimer’s disease: advances in immune-related blood biomarkers and computational approaches
Source: Front Immunol. 2024 Apr 23;15:1343900. doi: 10.3389/fimmu.2024.1343900 (PMC11078023; doi:10.3389/fimmu.2024.1343900)
Supplement: Supplementary file 1 [file DataSheet_1.docx]

***Supplementary Material***

Towards early diagnosis of Alzheimer's disease:

Advances in immune-related blood biomarkers and computational modeling approaches

Sophia Krix^1,2^, Ella Wilczynski^3^, Neus Falgàs^4^, Raquel Sánchez-Valle^4^, Eti Yoles^5^, Uri Nevo^3,6^, Kuti Baruch^5^, Holger Fröhlich^1,2,*^

*** Correspondence:** Holger Fröhlich: holger.froehlich@scia.fraunhofer.de

# Supplementary Figures and Tables


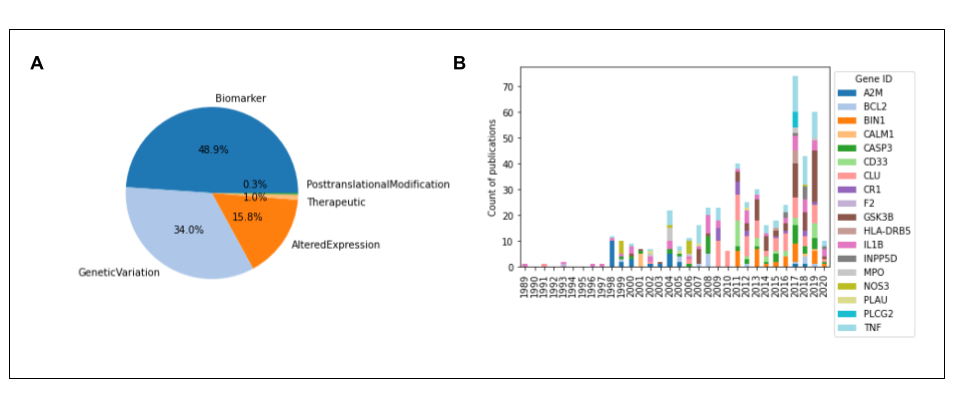
**Supplementary Figure 1.** Research findings identifying immune-system related genes in association with Alzheimer’s disease. We gathered all curated gene-disease associations from DisGeNet [(Piñero González et al., 2020)](https://www.zotero.org/google-docs/?EIN18i) for relevant immune system-related genes which we extracted from KEGG [(Kanehisa et al., 2023)](https://www.zotero.org/google-docs/?LI7Kjq). **A)** Proportional types of evidence for all curated entries in DisGeNet on Alzheimer’s disease associated with immune system-related genes. Entries in DisGeNet dating back to a final publication in the year 2000 mostly consist of biomarker discoveries, followed by findings on genetic variations and on altered expression. **B)** Detailed breakdown of number of biomarker publications per year on Alzheimer’s disease involving immune-system related genes by gene symbol. The number of research findings that associate immune system-related genes with Alzheimer’s disease has increased from 1990 - 2020. TNF, GSL3B and CLU are frequently appearing in recent publications on biomarkers in AD.

**Supplementary Table 1**. Traditional and potential future biomarkers for the diagnosis and staging of AD. The biological identification methods and the usefulness for staging or diagnosis of Alzheimer’s disease, as well as the capability for predicting progression from MCI to AD are listed with their corresponding publication. Biological identification methods are color-coded for CSF-based (blue), imaging (grey) and plasma-based (red) analyses. It is noteworthy that the assays listed below are predictive for different aspects of the pathology. Especially, the plasma tests for amyloid and tau measure the predictive performance of the test in terms of reported amyloid or tau burden, respectively. In the columns of diagnosis of clinical stages, a (+) sign indicates that the biomarker measured in the respective assay was able to predict the respective stage, in some cases in combination with other biomarker measures or patient genotypes, or the progression from MCI to AD, as was investigated in some studies. A (-) sign indicates that the prediction performance of the biomarker was not high enough for a precise diagnosis, and no sign ( ) indicates that this aspect was not part of the respective study.

| **Biomarker** | **Sample** | **Assay** | **Diagnosis of clinical stages** | | | | | **Used in publications** |
| --- | --- | --- | --- | --- | --- | --- | --- | --- |
|  |  |  | **MCI** | **PreAD** | **ProAD** | **AD-Dementia** | **MCI to AD** |  |
| **Aβ** | CSF | ELISA, Luminex Multiplex Assay | + | + | + | + |  | Jack et al., 2018 |
|  | Imaging | Amyloid-PET | + | + | + | + |  | Jack et al., 2018 |
|  | Blood | Mass spectrometry (PrecivityAD, C2N Diagnostics) | + |  |  | + |  | Monane et al., 2023  Hu et al., 2022 |
|  |  | CLEIA (HISCL series, Sysmex Corporation®) | + |  |  | + |  | Yamashita et al., 2022 |
| **pTau** | CSF | ELISA, Luminex Multiplex Assay | + | + | + | + |  | Jack et al., 2018 |
|  | Imaging | Tau-PET | + | + | + | + |  | Jack et al., 2018 |
|  | Blood | ECLIA (pTau 181 Elecsys® Amyloid Plasma Panel, Roche) | + |  |  | + | + | Palmqvist et al., 2022 |
|  |  | Simoa (pTau 181 and 231 assay,Quanterix, Billerica, MA, USA) | + |  |  | + |  | Doré et al., 2022 |
|  |  | ELISA (pTau 217 assay, ALZpath Inc.) |  | + | + | + |  | Ashton et al., 2024 |
|  |  | Mass spectrometry (pTau 217) | + |  |  | + |  | Barthelémy et al., 2024 |
|  |  | ELISA (brain-derived tau) |  |  |  | + |  | Gonzalez-Ortis et al., 2023 |
| **Neuro-**  **inflammation** | CSF | ELISA, Luminex Multiplex Assay | + | + | + | + |  | Jack et al. 2018 |
|  | Imaging | FDG-PET | + | + | + | + |  | Jack et al., 2018 |
|  | Blood | Simoa (Total tau, Quanterix, Billerica, MA, USA) | - |  |  | - | + | Mielke et al., 2017 |
| **sTREM2** | Blood | ELISA (RayBiotech/Abcam) | - |  |  |  | + | A. Zhao et al., 2022 |
|  |  | Luminex xMap | - |  |  | + |  | Weber et al., 2022 |
| **Galectin-3** | Blood | ELISA | - | - | - | + |  | Yazar et al., 2021 |
|  |  | ELISA (R&D Systems, Minneapolis, Minnesota) | - |  |  | + |  | Wang et al., 2013 |
| **GFAP** | Blood | Simoa (Quanterix®, Billerica, MA, USA) |  |  |  |  | + | Cicognola et al., 2021 |
|  |  |  | + | + | + | + |  | Pereira et al., 2021; Ferrari-Souza et al., 2022 |
|  |  |  | + |  |  | + |  | Bucci et al., 2023 |
|  |  | ECLIA (S-PLEX® assay kits, MSD, Rockville, MD, USA) |  |  |  | + | + | Kivisäkk et al., 2023 |
| **CHI3L1/YKL-40** | Blood | ELISA (Quidel, San Diego, California, USA) | - | - | - | - |  | Craig-Shapiro et al., 2010 |
|  |  |  |  |  |  | + |  | Villar-Piqué et al., 2019 |
| **PD-1** | Blood | Flow cytometry | + | + | + | + |  | Wu et al., 2022 |

**Supplementary Table 2.** Immune system-related blood biomarkers used in omics-based machine learning approaches for biomarker signature discovery in AD and MCI individuals. Listed are protein biomarkers that were found to distinguish AD from HC, AD from MCI and MCI from HC individuals. The protein name reported in the study is listed with its gene symbol in brackets. Biomarkers listed here are filtered for immune-system related biomarkers by their occurrence in experimentally validated interactions of the InnateDB [(Breuer et al., 2013)](https://www.zotero.org/google-docs/?ajqLoW), a manually curated knowledgebase of interactions involved in mammalian innate immunity.

| **Protein Biomarkers AD vs HC (Gene Symbol)** | **Protein Biomarkers AD vs MCI (Gene Symbol)** | **Protein Biomarkers MCI vs HC (Gene Symbol)** | **Reference** |
| --- | --- | --- | --- |
| C4 (C4A)  Eotaxin-1 (CCL11)  CR1 (CD1)  C5 (C5)  CRP (CRP) | Eotaxin-1 (CCL11)  MIP-1-Beta (CCL4)  FI (CFI)  C3 (C3)  CRP (CRP)  MCP-1 (CCL2) | FH (CFH)  C3 (C3)  MCP-1 (CCL2)  C5 (C5)  MIP-1-Beta (CCL4) | Morgan et al., 2019 |
| A2M (A2M)  MDC (CCL22)  IL-18 (IL18)  CD5L (CD5L) | - | - | Prabhakar & Bhargavi, 2022 |
| LRRIQ2 (CEP97)  CAMLG (CAMLG)  IL-4 (IL4)  TPM1 (TPM1)  IL-20 (IL20)  DIABLO (DIABLO)  VRK3 (VRK3) | - | - | Karaglani et al., 2020 |
| Eotaxin-3 (CCL26)  A1M (AMBP)  PLGF (PGF)  PYY (PYY)  Pancreatic Polypeptide (PPY)  A2M (A2M)  CRP (CRP) EGF (EGF) | - | - | Jammeh et al., 2016 |
| A1M (AMBP)  A2M (A2M)  C3 (C3)  TNC (TNC) | - | - | Eke et al., 2018 |
| CypA (PPIA)  HO-1 (HMOX1)  IRE1 (ERN1) | - | CypA (PPIA)  HO-1 (HMOX1)  IRE1 (ERN1) | Choi et al., 2021 |
| ALB (ALB)  DNAJB9 (DNAJB9)  FAP (FAP)  F8 (F8)  GABARAPL1 (GABARAPL1)  GRID2 (GRID2)  MMP12 (MMP12)  GHR (GHR)  GDF12 (GDF15) | - | - | Walker et al., 2023 |
| ABCA2 (ABCA2)  CREBRF (CREBRF)  CD72 (CD72)  CETN2 (CETN2)  KCNG1 (KCNG1)  NDUFA2 (NDUFA2) | - | - | Abdullah et al., 2022 |
| ANKRD28 (ANKRD28)  CCDC92 (CCDC92)  DEFA3 (DEFA3)  FBXO32 (FBXO32)  GRIA4 (GRIA4)  HDAC7 (HDAC7)  IFITM3 (IFITM3)  MC1R (MCRI)  RPL18 (RPL18)  SPOCD1 (SPOCD1)  ST14 (ST14)  TOR1AIP2 (TOR1AIP2)  TRIM16L (TRIM16L)  UBXN7 (UBXN7)  VEGFB (VEGFB) | - | - | Kim & Lee, 2022 |
| CFLAR,ALDH3B1,HFE,HGF,CDH1,PIK3CB,CASP8,RHOA,PRKCZ,CDC42,PRKACA,FCGR2B,GLI2,SRI,ARAF,DNM2,HADHA,BAX,DLD,SIRT1,ABL1,MAPK1,HMOX1,TSPO,ACO2,SOS2,MAPK3,NBN,IKBKB,AKT2,PIK3R2,MAPK8,PPARGC1A,IFNG,PPARD,MAPK14,BCL6,HES1,NFE2L2,PLA2G4A,AKT3,APOA1,MYB,PTK2B,PIK3CA,FLT3,C3,RAF1,PRKAA1,KRAS,CD36,TLR4,TLR2,ARRB1,THBS1,PLCB2,LEF1,PPP3CA,GATA6,APP,AKT1,SNCA,SRD5A1,PIK3R1,NOTCH1,TCF7L2,ATM,ADRA2A,BCL2L11,KIT,CBS,IL23R,PRKCB,NOD2,RBPJ,CX3CR1,MAP2K1,SOCS5,BCL2,PTEN,RELA,JUP,LEP,HRAS,GRB2,GATA2,PRKX,PLA2G6,HDAC2,ADA,SRC,PLCG2,BMPR2,HLA-A,NRAS,CHUK | - | - | Zhang et al., 2023 |
| MRPL10 (MRPL10)  SPG7 (SPG7)  MRPS25 (MRPS25)  NIT2 (NIT2)  OPA1 (OPA1)  DLD (DLD) | - | - | Liu, Li and Pan, 2021 |
| ROR (RORC)  GATA (GATA3)  IL4 (IL4)  IL17A (IL17A)  IL9 (IL9)  TNF-alpha (TNF)  IL21 (IL21)  IL10 (IL10)  IL6 (IL6)  IL23 (IL23) | (unsignificant) | GATA (GATA3)  IL4 (IL4)  IL17A (IL17A)  TNF-alpha (TNF)  IL21 (IL21)  IL10 (IL10)  IL6 (IL6)  IL23 (IL23) | Gironi et al., 2015 |
| MGC:31944 (IGLC2)  MGC:31936 (IGLL1)  MGC:27152 (IGLV2-23)  HSH2D (HSH2D)  CPSF3 (CPSF3)  C20orf96 (C20orf96)  SDF-1a (CXCL12) | MGC:31944 (IGLC2)  MGC:31936 (IGLL1)  MGC:27152 (IGLV2-23)  HSH2D (HSH2D)  CPSF3 (CPSF3)  C20orf96 (C20orf96)  SDF-1a (CXCL12) | MGC:31944 (IGLC2)  MGC:31936 (IGLL1)  MGC:27152 (IGLV2-23)  HSH2D (HSH2D)  CPSF3 (CPSF3)  C20orf96 (C20orf96)  SDF-1a (CXCL12) | De Marshall, 2016 |
| LGALS1 (LGALS1)  ICAM4 (ICAM4)  DNAJC8 (DNAJC8) | - | IGLC2 (IGLC2)  HSH2D (HSH2D)  GCDH (GCDH)  CCL19 (CCL19) | De Marshall, 2023 |

[Breuer, K., Foroushani, A. K., Laird, M. R., Chen, C., Sribnaia, A., Lo, R., Winsor, G. L., Hancock, R. E. W., Brinkman, F. S. L., & Lynn, D. J. (2013). InnateDB: Systems biology of innate immunity and beyond--recent updates and continuing curation. *Nucleic Acids Research*, *41*(Database issue), D1228-1233. https://doi.org/10.1093/nar/gks1147](https://www.zotero.org/google-docs/?prNArR)

[Kanehisa, M., Furumichi, M., Sato, Y., Kawashima, M., & Ishiguro-Watanabe, M. (2023). KEGG for taxonomy-based analysis of pathways and genomes. *Nucleic Acids Research*, *51*(D1), D587–D592. https://doi.org/10.1093/nar/gkac963](https://www.zotero.org/google-docs/?prNArR)

[Piñero González, J., Ramírez Anguita, J. M., Saüch Pitarch, J., Ronzano, F., Centeno, E., Sanz, F., & Furlong, L. I. (2020). *The DisGeNET knowledge platform for disease genomics: 2019 update*. https://doi.org/10.1093/nar/gkz1021](https://www.zotero.org/google-docs/?prNArR)
